# Adherence to Prescribing Indicators at a District Hospital in Ghana: Do We Match WHO Standards?

**DOI:** 10.3390/ijerph191912260

**Published:** 2022-09-27

**Authors:** Obed Kwabena Offe Amponsah, Nana Kwame Ayisi-Boateng, Sharath Burugina Nagaraja, Divya Nair, Karlos Muradyan, George Kwesi Hedidor, Appiah-Korang Labi, Mercy Naa Aduele Opare-Addo, Emmanuel Sarkodie, Kwame Ohene Buabeng

**Affiliations:** 1 Department of Pharmacy Practice, Kwame Nkrumah University of Science and Technology, Kumasi 00233, Ghana; 2 Department of Medicine, Kwame Nkrumah University of Science and Technology, Kumasi 00233, Ghana; 3 University Hospital, Kwame Nkrumah University of Science and Technology, Kumasi 00233, Ghana; 4 Department of Community Medicine, ESIC Medical College and PGIMSR, Bengaluru 560010, India; 5 International Union Against TB and Lung Disease (The Union), 75006 Paris, France; 6 Tuberculosis Research and Prevention Center, Yerevan 0014, Armenia; 7 WHO Country Office, Ghana, 7 Ameda Street, Roman Ridge, Accra P.O. Box MB 142, Ghana

**Keywords:** drug use review, outpatients, Ghana, SORT IT, quality indicators, electronic medical records, operational research, antimicrobial resistance, RUM, University hospital

## Abstract

**(1) Background**: Rational use of medicines (RUM) and their assessment are important to ensure optimal use of resources and patient care in hospitals. These assessments are essential to identifying practice gaps for quality improvement. **(2) Methods**: Assessment of adherence to WHO/International Network for Rational Use of Drugs core prescribing indicators among outpatients in 2021 was conducted at the University Hospital of the Kwame Nkrumah University of Science and Technology in the Ashanti Region of Ghana. We reviewed electronic medical records (EMR) of 110,280 patient encounters in the year which resulted in 336,087 medicines prescribed. **(3) Results**: The average number of medicines prescribed per encounter was three, with generics being prescribed in 76% of prescriptions. Injections were prescribed in 7% of encounters while 90% of medicines were from Ghana’s Essential Medicines List, 2017. **(4) Conclusions**: With the exception of patient encounters with injections, none of the prescribing indicators assessed in this study met WHO optimum levels, providing targets for quality improvement in RUM. Implementing prescribing guides and policies, regular audits and feedback as well as continuous professional development training may help to improve prescribing practices in the hospital.

## 1. Introduction

Rational use of medicines (RUM) in hospitals and related institutions is important to ensure patients obtain the best care possible. It is also essential for ensuring that healthcare delivery is optimal, and resources are used in a prudent manner. The World Health Organization (WHO) defines rational use of medicines as “patients receiving medications appropriate to their clinical needs, in doses that meet their own individual requirements, for an adequate period of time, and at the lowest cost to them and their community” [1]. A previous report from WHO showed that half of medicines across the world are prescribed or sold inappropriately and that half of patients are unable to use their medicines correctly, which is a matter of concern [2]. The World Bank estimates that medicines account for 20–50% of all healthcare expenses in low-and-middle-income countries (LMIC) [3]. RUM is especially important in LMICs such as Ghana that are constrained in terms of resources and effective regulation of supply and use of medications [4].

Inappropriate use of medicines is associated with increased mortality and morbidity caused by chronic medical conditions such as diabetes, hypertension and neurological disorders [5]. An example of irrational prescribing leading to poor clinical outcomes is that of antimicrobial prescribing, which is associated with increasing antimicrobial resistance (AMR) and an attendant poor patient outcome [6]. For instance, in Ghana and around the world, studies have reported a high use of antibiotics (34.4–60.5%) in different settings [7,8,9]. A study from Uganda reported worsened patient outcomes and increased mortality of 20% associated with irrational antibiotic use [10].

The WHO in a collaborative effort with the International Network for Rational Use of Drugs (INRUD) has developed a set of indicators to measure hospital performance in relation to medicine use. These indicators serve as a tool to monitor and guide the health care services for proper documentation of medicine usage with the emphasis on prescribing practices, patient care and facility specific factors [11]. These indicators include standardized core medicine prescribing indicators to ensure rational use of medicines (RUM) [12]. Each indicator has been assigned an optimum score to allow objective assessment and promote RUM [13]. The prescribing indicators provide basic information on drug prescribing practices in a hospital. Assessing the quality of prescribing in hospitals is key to identifying gaps in practice and opportunities for quality improvement.

A recent assessment of outpatient antibiotic prescribing at the University Hospital, Kwame Nkrumah University of Science and Technology (KNUST) found antibiotic prescribing to be high at 36% (WHO optimum: 20–26.8%), indicating the need for standard guidelines as part of outpatient antimicrobial stewardship (AMS) [14]. To maintain high quality patient care and disease pharmacotherapy, it is prudent to assess the prescribing practices of prescribers in the University Hospital of KNUST. This will help in the investigation of the prevalent practices which will benefit from quality improvement of outpatient care at the hospital. Additionally, in Ghana, no studies have been reported on RUM in the Ashanti region which is the second largest administrative region.

We conducted a study to assess adherence to the WHO/INRUD rational use of medicines prescribing indicators at the outpatient department of University Hospital, KNUST in the Ashanti region of Ghana in 2021. The specific objectives were to assess: (a) average number of medicines prescribed per patient encounter; (b) the number and proportion of medicines prescribed by generic names; (c) the number and proportion of patient encounters with an injection prescribed and (d) the number and proportion of medicines prescribed from Ghana’s Essential Medicines List (EML), 2017.

## 2. Materials and Methods

### 2.1. Study Design 

A cross-sectional study was conducted using routinely collected electronic data from outpatient medical records of the University Hospital, KNUST.

### 2.2. Settings 

#### General Setting

Ghana is a country in West Africa with a population of 30.8 million as of 2021. The country is divided into 16 regions with Accra being the capital city [15]. The healthcare services in Ghana are mainly organized through a three-tiered system (primary, secondary and tertiary). Health services in Ghana are provided mainly by public hospitals (54%), private hospitals (40%) and mission hospitals (6%) [16]. Some of the public hospitals are classified as quasi-governmental hospitals because they are funded by both the government and the private sector. 

### 2.3. Specific Settings

The University Hospital, KNUST, is a quasi-governmental district-level hospital with an inpatient capacity of 135 beds and has a patient foot fall of almost 100 patients per day at the outpatient department (OPD). The hospital is centrally located in the Oforikrom municipality of the Ashanti region of Ghana [15]. The hospital serves the university community and a catchment area of 303,016 people in the municipality [17]. The hospital provides a range of general and specialist care services to patients.

#### 2.3.1. Outpatient Department of the University Hospital, KNUST 

The OPD of the University Hospital is the first port of call for patients presenting to the hospital. The OPD is manned by physicians and physician assistants who evaluate all patients and decide on whether outpatient or inpatient care is warranted. All the patient details are entered into the electronic medical records of the hospital.

#### 2.3.2. Outpatient Medical Records

The hospital uses an electronic medical record (EMR) system to document patient records. The system has various levels of modules to facilitate the documentation of patient care including a prescriber consultation module, a laboratory information system module, a pharmacy module and an inventory module, all of which are linked in one way or another to improve care.

### 2.4. Study Population 

The study included medicines prescribed for all patients managed at the OPD of the University Hospital between January and December 2021. 

### 2.5. Data Variables and Analysis

Data on demographic and clinical characteristics and drug prescriptions of patients were retrieved from the EMR system of the OPD. Data from the EMR were retrieved in a MS Excel CSV format, cleaned and processed using Python in Jupyter Notebook, a web-based interactive computing platform [18], and analyzed using STATA^®^ (version 16.0 Copyright 1985–2019 StataCorp LLC, College Station, TX, USA). 

The prescribing indicators developed by the WHO/INRUD were assessed in this study, with the exception of encounters with antibiotics prescribed, which have already been assessed [14]. Based on the available data variables [12], we calculated the following indicators and summarized in Table 1:

1. Average number of medicines per encounter;
2. Number and proportion of medicines prescribed by generic names;
3. Number and proportion of encounters with an injection prescribed;
4. Number and proportion of medicines prescribed from the Essential Medicines List.

Indicator 1 is summarized as an average with its range. Indicators 2, 3 and 4 are summarized as percentages with their 95% confidence interval (95% CI).

## 3. Results

In total, 350,149 prescriptions were given in 110,280 patient encounters out of which 336,087 were medicines that were included in the study. 

### 3.1. Medicines Prescribed per Patient Encounter

Overall, an average number of 3 medicines were prescribed per patient encounter in 2021 with a range of 1 to 18 medicines. July and December had the highest average number of medicines prescribed at 3.3 medicines per encounter, while January had the lowest average number of medicines prescribed at 2.8. Table 2 shows the monthly distribution of medicines prescribed at the outpatient department in 2021.

### 3.2. Medicines Prescribed in Generic Format

Of a total 335,968 medicines prescribed in 2021, 253,987 accounting for 76% (95% CI: 75.4–75.7%) were prescribed in their international non-proprietary name (INN) or generic name. Across the year, prescribing of medicines in their INN ranged from 74% to 77% monthly (Table 3).

### 3.3. Prescriptions with Injections

Of all (110,280) patient encounters in the year, 7608 (6.8%, 95% CI: 6.74–7.04%) contained injections. Each month in 2021, the proportion of encounters with injections prescribed ranged from 6% to 8% (Table 4).

### 3.4. Medicines Prescribed from the Essential Medicines List

Of 335,968 medicines prescribed, 302,319 (90%, 95% CI: 89.8–90.1%) were from Ghana’s Essential Medicines List (EML). On a monthly basis, prescribing from the EML ranged from 89% to 91% of medicines prescribed (Table 5).

## 4. Discussion

This is one of the few studies on prescription practices conducted in Ghana and our study findings reveal that the prescribing practices at the study site failed to meet the benchmark of WHO standards. The prescribing indicators assessed provided a baseline indication of prescribing practices among outpatients at the hospital. The implications of the findings are discussed below.

(a.) Average number of medicines per patient encounter: There was a high number of medicines prescribed per patient encounter, beyond the WHO optimum of less than two (2). The study finding is also higher than that reported from other regions including Africa (2.6–3.1), the Americas (1.8), Europe and Southeast Asia (2.5) [19,20]. Findings from Tanzania and Pakistan were also lower at 2.3 and 2.8, respectively [21,22]. Our findings were, however, lower than that previously found in Sierra Leone (4.18–4.56) and another study from northern Ghana (3.9) [23,24]. All of these studies used a sample of prescriptions for assessment, unlike this study which included all patient encounters which had medicines prescribed. This indicator provides basic information on polypharmacy which has been associated with adverse patient outcomes and medication errors. A previous study demonstrated a correlation between adverse drug events and the number of medicines prescribed while another found that patients were less likely to remember medicine schedules with higher numbers of medicines [25,26]. The results could be due to the absence of local practice guidelines in the hospital. The high average number of medicines could be improved through the use of clinical practice guidelines in the hospital. This could be achieved through adaptation of evidence-based guidelines to the local context to improve prescribing:

(b.) Percentage of medicines prescribed by generic name: Prescribing by generic or INN name in the hospital was a third less than WHO’s optimum of 100%. This may be a target for quality improvement in prescribing practices among outpatients in the hospital. The results of this study are also much lower than findings from Tanzania (95.7%) and Ethiopia (89.13–97.96%), but higher than studies from Pakistan (56.6%), Sierra Leone (57%) and Ghana (53%) [21,22,23,24,27]. The varied findings could be as a result of the fact that all the studies included prescriptions from multiple health facilities, providing a possible buffering effect on the average scores. However, this study may be a call to action in the University Hospital, KNUST to put in place measures to increase prescribing by generic names of medicines. This is due to the relative low cost of generic medicines compared to branded medicines. This makes it easier for patients to access medicines which may improve adherence to therapy in light of economic difficulties [28,29]. A previous assessment of medicine prices in Ghana found that branded medicines were much more expensive than generic medicines. For instance, management of a peptic ulcer using branded medicines cost 86.6 days’ wages compared to 10.9 days’ wages for treatment with generic equivalents which are widely available [30]. In LMICs, it is important to ensure that patients are not skipping medicines due to high costs but rather saving costs through generic medicine use [31]. To improve the prescribing of medicines by generic names, prescriber sensitization and training through continuous professional development could help to increase rational prescribing in the hospital. Restrictive prescribing policies and guides could also potentially improve prescribing by generic names [32]. Performing an economic assessment of medicine costs borne by patients in the hospital from generic and branded medicines may provide objective information to advise policy and practice. A qualitative study to understand why prescribers are prescribing branded medicines may be useful in identifying measures to reverse the current trend;

(c.) Percentage of encounters with an injection prescribed: Patient encounters with an injection prescribed were 13% lower than the WHO optimum of less than 20%. Across Africa, patient encounters with injections prescribed ranged on average from 14% to 57.6% in previous studies [24,33], while one study from Pakistan had no injections prescribed [22]. This is commendable as the increased use of injections which require invasive devices, such as peripheral vascular catheters and needles, may increase the risk for blood-borne infections [34,35,36]. Reduced use could be maintained through audit and feedback to prescribers on prescribing patterns; 

(d.) Percentage of medicines prescribed from an essential medicines list: Overall, the proportion of medicines prescribed from Ghana’s Essential Medicines List (EML) [37] was 10% less than the WHO optimum of 100%. This is higher than that found in Sierra Leone (64%) and the average in the WHO African region (88%) [20,23]. Other studies have, however, reported prescribing from EMLs higher than this study—between 92.54% and 98.8% [21,22,24,27]. This may indicate a relatively high level of prescribing from the EML in the hospital, with room for improvement. Adherence to prescribing from the EML is a key tool for ensuring stable healthcare delivery in countries. This ensures that quality medicines are available and affordable across the board, thus promoting RUM [38,39]. To improve prescribing from the EML, the previously suggested prescribing guides and policies could include a policy on widespread prescribing from the EML to ensure RUM. Continuous prescription audits and feedback to prescribers could also help improve prescribing in the hospital.

The EMR of the hospital facilitated the audit of prescribing patterns in the hospital to identify targets for quality improvement. This allowed relatively easy access to data, allowing inclusion of all data for analyses, unlike other studies which only sampled prescriptions from patient encounters. Other hospitals in Ghana will likely benefit from such use of technology to ensure rapid audit and feedback to prescribers and hospitals to improve RUM.

The study has the following strengths. First is the large dataset from the EMR which was available and used for analyses. Second, the completeness of study variables used for this study was high at approximately 98%. Third, the study was conducted and reported in accordance with the Strengthening the Reporting of Observational Studies in Epidemiology (STROBE) guidelines statement [40]. There are few limitations of the study. The data were from only one hospital which may limit the general applicability of the study findings. Additionally, comparison between visits made by individual patients at different times during the study period could not be carried out, as the database has a limitation of not being able to ascertain if a subsequent visit has been made by the same patient for the same or similar complaint

## 5. Conclusions

The hospital practices of RUM were mostly not in accordance with the WHO/INRUD standards. However, the use of injections seemed to be compliant with the standards. There is a huge potential for implementing prescribing guides and policies, undertake regular audits and feedback as well as continuous professional development for improvement in the quality of medicine prescribing, improved care and outcomes in the hospital.

## Figures and Tables

**Table 1:** Data variables for assessing prescribing indicators among outpatients at University Hospital, KNUST in 2021.

INDICATOR	NUMERATOR	DENOMINATOR	WHO OPTIMUM [12]
Average number of medicines per patient encounter	Total number of medicines prescribed	Total patient encounters for which data were collected	<2
Percentage of medicines prescribed by generic name	Total number of medicines prescribed in INN format	Total number of medicines prescribed	100%
Percentage of encounters with an injection prescribed	Number of encounters in which an injectable form of medicine was prescribed	Total patient encounters for which data were collected	<20%
Percentage of medicines prescribed from an essential medicines list	Total number of medicines prescribed from the EML	Total number of medicines prescribed	100%

INN—International Non-proprietary Name; KNUST—Kwame Nkrumah University of Science and Technology.

**Table 2:** Average number of medicines prescribed per patient encounter among outpatients at University Hospital, KNUST in 2021.

Month	Total Patient Encounters for Which Data were Collected	Total Number of Medicines Prescribed	Average Number of Medicines per Encounter	Lowest Number of Medicines Prescribed in an Encounter	Highest Number of Medicines Prescribed in an Encounter
January	8488	23,652	2.8	1	12
February	9406	29,794	3.2	1	13
March	10,338	30,541	3	1	13
April	8688	24,840	2.9	1	15
May	8800	26,634	3	1	14
June	11,679	35,538	3	1	15
July	13,339	44,506	3.3	1	18
August	10,640	31,262	2.9	1	15
September	8343	24,740	3	1	16
October	6864	21,144	3.1	1	13
November	6421	19,115	3	1	15
December	7274	24,321	3.3	1	14
Total	110,280	336,087	3	1	18

KNUST—Kwame Nkrumah University of Science and Technology.

**Table 3:** Number and proportion of medicines prescribed in the INN format among outpatients of University Hospital, KNUST in 2021.

Month	Total Number of Medicines Prescribed ^1^	Total Number of Medicines Prescribed in INN Format	Proportion of Medicines Prescribed by Generic Names
January	23,648	18,253	77%
February	29,789	23,022	77%
March	30,529	23,267	76%
April	24,832	18,674	75%
May	26,620	19,851	75%
June	35,516	26,195	74%
July	44,490	34,146	77%
August	31,251	23,831	76%
September	24,733	18,607	75%
October	21,137	15,909	75%
November	19,111	14,325	75%
December	24,312	17,907	74%
Total	335,968	253,987	76%

^1^ Denominator is the total number of medicines prescribed excluding any prescription which did not contain any drugs, or prescriptions which contained medical devices; INN—International Non-proprietary Name; KNUST—Kwame Nkrumah University of Science and Technology.

**Table 4:** Number and proportion of patient encounters with injections prescribed among outpatients of University Hospital, KNUST in 2021.

Month	Total Patient Encounters for Which Data Were Collected	Total Number of Encounters with an Injection Prescribed	Proportion of Encounters with an Injection Prescribed
January	8488	511	6%
February	9406	658	7%
March	10,338	658	6%
April	8688	588	7%
May	8800	569	6%
June	11,679	826	7%
July	13,339	927	7%
August	10,640	765	7%
September	8343	633	8%
October	6864	446	6%
November	6421	419	7%
December	7274	608	8%
Total	110,280	7608	7%

KNUST—Kwame Nkrumah University of Science and Technology.

**Table 5:** Number and proportion of medicines prescribed from the Ghana Essential Medicines List among outpatients of the University Hospital, KNUST in 2021.

Month	Total Number of Medicines Prescribed ^1^	Total Number of Medicines Prescribed from EML	Proportion of Medicines Prescribed from EML
January	23,648	21,461	91%
February	29,789	27,039	91%
March	30,529	27,491	90%
April	24,832	22,353	90%
May	26,620	23,623	89%
June	35,516	31,651	89%
July	44,490	39,830	90%
August	31,251	28,228	90%
September	24,733	22,401	91%
October	21,137	19,181	91%
November	19,111	17,089	89%
December	24,312	21,972	90%
Total	335,968	302,319	90%

^1^ Denominator is the total number of medicines prescribed excluding any prescription which did not contain any drugs or prescriptions which contained medical devices; EML–Essential Medicines List; KNUST–Kwame Nkrumah University of Science and Technology.

## Data Availability

Requests to access these data should be sent to the corresponding author.

## References

[1] World Health Organization The Rational Use of Drugs and WHO. https://apps.who.int/iris/bitstream/handle/10665/120708/em_rc33_r10_en.pdf.

[2] World Health Organization Promoting Rational Use of Medicines: Core Components. https://apps.who.int/iris/bitstream/handle/10665/67438/WHO_EDM_2002.3.pdf.

[3] Govindaraj R., Reich M.R., Cohen J.C. World Bank Pharmaceuticals. License: CC BY 3.0 IGO. https://openknowledge.worldbank.org/handle/10986/13734.

[4] Haque M. (2017). Antimicrobial Use, Prescribing, and Resistance in Selected Ten Selected Developing Countries: A Brief Overview. Asian J. Pharm. Clin. Res..

[5] O’Connor M.N., Gallagher P., Omahony D. (2012). Inappropriate Prescribing: Criteria, Detection and Prevention. Drugs Aging.

[6] Davies J. (2006). Where Have All the Antibiotics Gone?. Can. J. Infect. Dis. Med. Microbiol..

[7] Klein E.Y., Van Boeckel T.P., Martinez E.M., Pant S., Gandra S., Levin S.A., Goossens H., Laxminarayan R. (2018). Global Increase and Geographic Convergence in Antibiotic Consumption between 2000 and 2015. Proc. Natl. Acad. Sci. USA.

[8] Amponsah O.K.O., Buabeng K.O., Owusu-Ofori A., Ayisi-Boateng N.K., Hämeen-Anttila K., Enlund H. (2021). Point Prevalence Survey of Antibiotic Consumption across Three Hospitals in Ghana. JAC-Antimicrobial Resist..

[9] Versporten A., Zarb P., Caniaux I., Gros M.F., Drapier N., Miller M., Jarlier V., Nathwani D., Goossens H., Koraqi A. (2018). Antimicrobial Consumption and Resistance in Adult Hospital Inpatients in 53 Countries: Results of an Internet-Based Global Point Prevalence Survey. Lancet Glob. Health.

[10] Abeja C.J., Niyonzima V., Byagamy J.P., Obua C. (2022). Antibiotic Prescription Rationality and Associated In-Patient Treatment Outcomes in Children under-Five with Severe Pneumonia at Bwizibwera Health Center IV, Mbarara District, South-Western Uganda. Pneumonia.

[11] World Health Organization Action Programme on Essential Drugs and Vaccines How to Investigate Drug Use in Health Facilities: Selected Drug Use Indicators. https://apps.who.int/iris/handle/10665/60519.

[12] Ofori-Asenso R. (2016). A Closer Look at the World Health Organization’s Prescribing Indicators. J. Pharmacol. Pharmacother..

[13] Isah A., Laing R., Quick J., Mabadeje A.F.B., Santoso B., Hogerzeil H., Ross-Degnan D. (2001). The Development of Reference Values for the WHO Health Facility Core Prescribing Indicators. West Afr. J. Pharmacol. Drug Res..

[14] Amponsah O.K.O., Nagaraja S.B., Ayisi-Boateng N.K., Nair D., Muradyan K., Asense P.S., Wusu-Ansah O.K., Terry R.F., Khogali M., Buabeng K.O. (2022). High Levels of Outpatient Antibiotic Prescription at a District Hospital in Ghana: Results of a Cross Sectional Study. Int. J. Environ. Res. Public Health.

[15] 2021 Population and Housing Census—Ghana Statistical Service. https://census2021.statsghana.gov.gh/.

[16] Ghana Hospitals Categories. https://www.ghanahospitals.org/categories/.

[17] Ministry of Finance G. Composite Budget for 2020–2023 Programme Based Budget Estimates for 2020 Oforikrom Municipal Assembly. https://www.mofep.gov.gh/sites/default/files/composite-budget/2020/AR/Oforikrom.pdf.

[18] Kluyver T., Ragan-Kelley B., Pérez F., Granger B., Bussonnier M., Frederic J., Kelley K., Hamrick J., Grout J., Corlay S. (2016). Jupyter Notebooks-a Publishing Format for Reproducible Computational Workflows.

[19] Shankar P.R. (2009). Medicines Use in Primary Care in Developing and Transitional Countries: Fact Book Summarizing Results from Studies Reported between 1990 and 2006. Bull. World Health Organ..

[20] Ofori-Asenso R., Brhlikova P., Pollock A.M. (2016). Prescribing Indicators at Primary Health Care Centers within the WHO African Region: A Systematic Analysis (1995–2015). BMC Public Health.

[21] Irunde H., Minzi O., Moshiro C. (2017). Assessment of Rational Medicines Prescribing in Healthcare Facilities in Four Regions of Tanzania. J. Pharm. Pract. Community Med..

[22] Atif M., Sarwar M.R., Azeem M., Umer D., Rauf A., Rasool A., Ahsan M., Scahill S. (2016). Assessment of WHO/INRUD Core Drug Use Indicators in Two Tertiary Care Hospitals of Bahawalpur, Punjab, Pakistan. J. Pharm. Policy Pract..

[23] Cole C.P., Routledge P. (2018). An Evaluation of Rational Prescribing in Hospital Outpatient Practice in Sierra Leone and Assessment of Affordability of a Prescription as an Outcome. Pan Afr. Med. J..

[24] Baba S.M., Salifu A.T. (2019). Medicines Prescribing Pattern in Northern Ghana: Does It Comply with WHO Recommendations for Prescribing Indicators?. Afr. J. Pharm. Pharmacol..

[25] Kapp P.A., Klop A.C., Jenkins L.S. (2013). Drug Interactions in Primary Health Care in the George Subdistrict, South Africa: A Cross-Sectional Study. S. Afr. Fam. Pract..

[26] Uzochukwu B.S.C., Onwujekwe O.E., Akpala C.O. (2002). Effect of the Bamako-Initiative Drug Revolving Fund on Availability and Rational Use of Essential Drugs in Primary Health Care Facilities in South-East Nigeria. Health Policy Plan..

[27] Tefera B.B., Getachew M., Kebede B. (2021). Evaluation of Drug Prescription Pattern Using World Health Organization Prescribing Indicators in Public Health Facilities Found in Ethiopia: Systematic Reviews and Meta-Analysis. J. Pharm. Policy Pract..

[28] King D.R., Kanavos P. (2002). Encouraging the Use of Generic Medicines: Implications for Transition Economies. Croat. Med. J..

[29] Shrank W.H., Hoang T., Ettner S.L., Glassman P.A., Nair K., DeLapp D., Dirstine J., Avorn J., Asch S.M. (2006). The Implications of Choice: Prescribing Generic or Preferred Pharmaceuticals Improves Medication Adherence for Chronic Conditions. Arch. Intern. Med..

[30] Ministry of Health Ghana Medicine Prices in Ghana: A Comparative Study of Public, Private and Mission Sector Medicine Prices. https://haiweb.org/wp-content/uploads/2015/07/Ghana-Report-Pricing-Surveys.pdf.

[31] Cameron A., Laing R. Cost Savings of Switching Private Sector Consumption from Originator Brand Medicines to Generic Equivalents. https://www.who.int/healthsystems/topics/financing/healthreport/35MedicineCostSavings.pdf.

[32] Godman B., Bishop I., Finlayson A.E., Campbell S., Kwon H.Y., Bennie M. (2013). Reforms and Initiatives in Scotland in Recent Years to Encourage the Prescribing of Generic Drugs, Their Influence and Implications for Other Countries. Expert Rev. Pharm. Outcomes Res..

[33] Ofori-Asenso R., Agyeman A.A. (2015). A Review of Injection and Antibiotic Use at Primary Health Care (Public and Private) Centers in Africa. J. Pharm. Bioallied Sci..

[34] Zhang L., Cao S., Marsh N., Ray-Barruel G., Flynn J., Larsen E., Rickard C.M. (2016). Infection Risks Associated with Peripheral Vascular Catheters. J. Infect. Prev..

[35] Sato A., Nakamura I., Fujita H., Tsukimori A., Kobayashi T., Fukushima S., Fujii T., Matsumoto T. (2017). Peripheral Venous Catheter-Related Bloodstream Infection Is Associated with Severe Complications and Potential Death: A Retrospective Observational Study. BMC Infect. Dis..

[36] Mermel L.A. (2017). Short-Term Peripheral Venous Catheter–Related Bloodstream Infections: A Systematic Review. Clin. Infect. Dis..

[37] Ghana National Drugs Programme Essential Medicines List. https://www.moh.gov.gh/wp-content/uploads/2020/07/GHANA-EML-2017.pdf.

[38] Aitken M. Understanding the Role and Use of Essential Medicines Lists. https://www.ifpma.org/resource-centre/understanding-the-role-and-use-of-essential-medicines-lists/.

[39] Atif M., Malik I., Dawoud D., Gilani A., Ahmed N., Babar Z.U.D. (2019). Essential Medicine List, Policies, and the World Health Organization. Encyclopedia of Pharmacy Practice and Clinical Pharmacy.

[40] Von Elm E., Altman D.G., Egger M., Pocock S.J., Gøtzsche P.C., Vandenbrouckef J.P. (2007). The Strengthening the Reporting of Observational Studies in Epidemiology (STROBE) Statement: Guidelines for Reporting Observational Studies. Bull. World Health Organ..

